# Electrical stimulation towards melanoma therapy via liquid metal printed electronics on skin

**DOI:** 10.1186/s40169-016-0102-9

**Published:** 2016-06-23

**Authors:** Jun Li, Cangran Guo, Zhongshuai Wang, Kai Gao, Xudong Shi, Jing Liu

**Affiliations:** Department of Dermatology, Peking Union Medical College Hospital, Chinese Academy of Medical Sciences and Peking Union Medical College, Beijing, 100730 People’s Republic of China; Department of Biomedical Engineering, School of Medicine, Tsinghua University, Beijing, 100084 People’s Republic of China; Institute of Laboratory Animal Sciences, Chinese Academy of Medical Sciences and Peking Union Medical College, Beijing, 100021 People’s Republic of China

**Keywords:** Melanoma therapy, Liquid metal electrode, Skin soft electronics, Tumor treating fields, Electrical therapy

## Abstract

**Background:**

We proposed a method of using electrical stimulation for treatment of malignant melanoma through directly spray-printing liquid metal on skin as soft electrodes to deliver low intensity, intermediate frequency electric fields.

**Methods:**

With patterned conductive liquid metal components on mice skin and under assistance of a signal generator, a sine wave electrical power with voltage of 5 V and 300 kHz could be administrated on treating malignant melanoma tumor.

**Findings:**

The experiments demonstrated that tumor volume was significantly reduced compared with that of the control group. Under the designed parameters (signal: sine wave, signal amplitude Vpp: 5 V and Vpp: 4 V, frequency: 300 kHz) of Tumor treating fields (TTFields) with the sprayed liquid metal electrode, four mice tumor groups became diminishing after 1 week of treatment. The only device-related side effect as seen was a mild to moderate contact dermatitis underneath the field delivering electrodes. The SEM images and pathological analysis demonstrated the targeted treating behavior of the malignant melanoma tumor. Further, thermal infrared imaging experiments indicated that there occur no evident heating effects in the course of treatment. Besides, the liquid metal is easy to remove through medical alcohol.

**Conclusions:**

Tumor treating fields through liquid metal electrode could offer a safe, straightforward and effective treatment modality which evidently slows down tumor growth in vivo. These promising results also raised the possibility of applying spray-printing TTFields as an easy going physical way for future cancer therapy.

## Background

As living cells consist of ions, polar or charged molecules, membranes and organelles, they are responsive to and often generate electric fields and currents. The electric activity of cells plays a key role in many essential biological processes. Low intensity, intermediate frequency, electric fields have been proved to inhibit cancerous cell growth in vitro by an anti-microtubule mechanism of action. However, the classical electrical treatment devices are generally rigid circuit microelectronics which may sometimes encounter troubles such as poor coupling performance and large contact comfortableness etc. Under the huge contact resistance thus induced between circuits and skin, the nearby tissues may easily subject to hurt due to extremely high electrical voltage. On the other hand, such electrodes may cause discomfort and even hurt to human body due to their mechanically rigid fixing property. Recently, the flexible electronics (electrical or composite inks) is increasingly investigated and gradually applied to bio-electronic measurement [1–5]. However, a tough issue lying behind such composite inks [6–8] is that they rely heavily on the high temperature annealing or other more complex process such as evaporation. Besides, these electronic inks’ conductive performance is still not good enough for practical use. As an alternative, the recently emerging room temperature liquid metal is easy to manufacture electronics directly on flexible substrates even skin with unique convenience [9–14]. However, most of the currently available printing methods could not fabricate the electrical components on skin, whose geometry is generally irregular and not easy to deposit a circuit. Recently, we proposed an efficient approach of using the atomized spraying method to directly print the room temperature liquid metal to fabricate electronics on skin at room temperature which does not request a thermal annealing process [15]. Here, we extend this technology into a quick and direct manufacturing tool to pattern electrical components on skin to tackle tumor diseases for the first time.

## Methods

### Treatment method

**Fig. 1:**
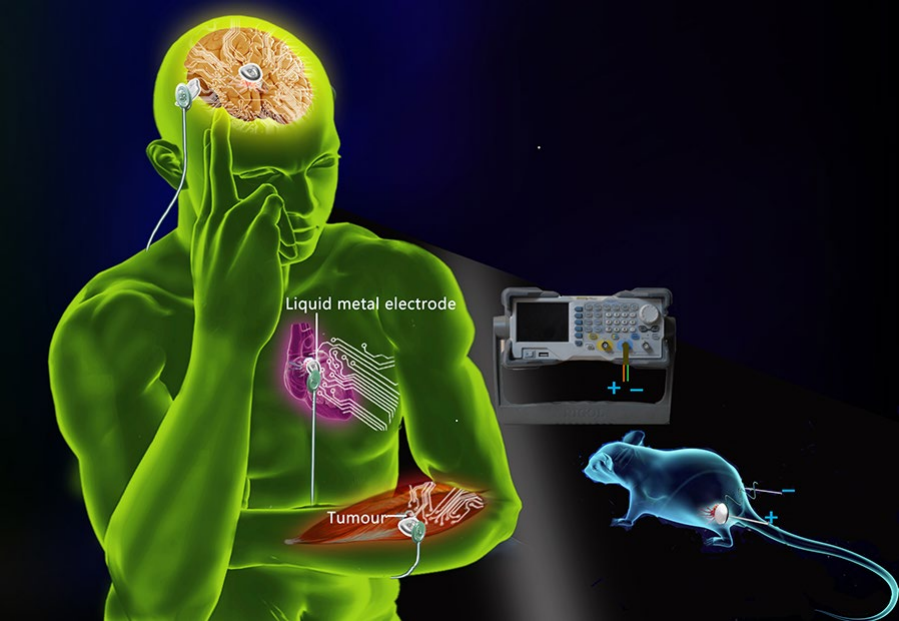
Principle of using liquid metal electrodes to deliver electrical stimulation for tumor therapy

According to Fig. 1, the current therapy for treating malignant melanoma tumor depends on the soft liquid metal electrodes and electrical fields delivered into the target site. To test the effectiveness of our method in destroying tumor cells in vivo, an animal tumor model of C57BL/6 mice inoculated intradermally with malignant melanoma cells (B16F10) was particularly adopted. Tumor treating fields (TTField) was administrated by means of 15-mm-long insulated wire needle (outer diameter, 0.3 mm; insulation thickness, 0.1 mm; Tefzel) inserted intradermally on one side of the tumor, then liquid metals were sprayed on the surface of the tumor, leaving only 4 cm^2^ normal skin on the other side of the tumor. Another identical wire needle was inserted intradermally into the 4 cm^2^ normal skin left on the other side of the tumor. TTFields were generated between liquid metals plus inserted (intradermal) wholly insulated wire needle placed on one side of the tumor and inserted (intradermal) wholly insulated wire needle placed on the other side of the tumor. Mice with inserted electrodes were treated 90 min per day for 6 days beginning 1 week after cell inoculation. The animal study has been approved by the Ethics Committee of Tsinghua University, Beijing, China under contract [SYXK (Jing) 2009-0022].

### Experimental design

Eight healthy C57/BL mice (Purchased from Beijing Weitong Lihua experimental animal center) were tested, whose subcutaneous skin were inoculated with melanoma B16 tumor cell lines.

After 14 days, the mice were divided into two groups: six mice in the experimental group and two mice in the control group. The experimental group was divided into three experimental groups with different test conditions: (A) Electrical stimulation parameters of mice model (signal: sine wave, signal amplitude Vpp: 5 V, frequency: 300 kHz); (B) (signal: sine wave, signal amplitude Vpp: 5 V, frequency: 100 kHz) and (C) (signal: sine wave, signal amplitude Vpp: 4 V, frequency: 300 kHz) respectively. Each group has 2 mice. The mice in the control group were sprayed with liquid metal electrodes on the surface of the tumor, leaving only 4 cm^2^ normal skin on the other side of the tumor without TTFields. The sine wave signal of TTFields through the electrode was administrated by means of 15-mm-long insulated wire needle. Mice were treated with TTFields 90 min per day for 6 days.

In the control group, the mice were also sprayed with liquid metals electrodes, with the same time length and interval of treatment without TTFields. Percutaneous measurements were made on tumor volume in eight mice, in the treatment of the beginning of the first 1d, 2d, 6d and 7d. Tumor volume can be calculated via formula: V = (*a* × *b*^2^)/2, where a is the longest diameter of the tumor and b is the longest perpendicular diameter length of a.

After 6 days of complete electrical stimulation treatment, in each group a mouse was sacrificed. The tumor tissues were removed and fixed with 10 % formalin for histopathological examination. Magnetic resonance imaging (MRI) examination was performed 24 h after the TTFields treatment with sprayed liquid metals electrodes removed by ethanol.

## Results

The average volume of the three experimental groups and control group tumor are presented in the following Table 1.

**Table 1:** Comparison of tumor volume between experimental group and control group after TTfields was applied (Mean value ± standard deviation)

Group	Days after the electric field applies (d) 1 2 6 7			
(A)	305.92 ± 90.88	98.5 ± 37.12	_*	_*
(B)	1567.60 ± 595.60	10,518.40 ± 8331.8	21,445.31 ± 708.51	22,803.63 ± 894.53
(C)	5147.27 ± 2984.07	3515.92 ± 2100.8	_*	_*
(Control)	1714.78 ± 228.72	6530.90 ± 875.10	7916.85 ± 1145.65	11,512.3 ± 1541.3

_*:Tumor is too small to measure

### Treatment outputs

**Fig. 2:**
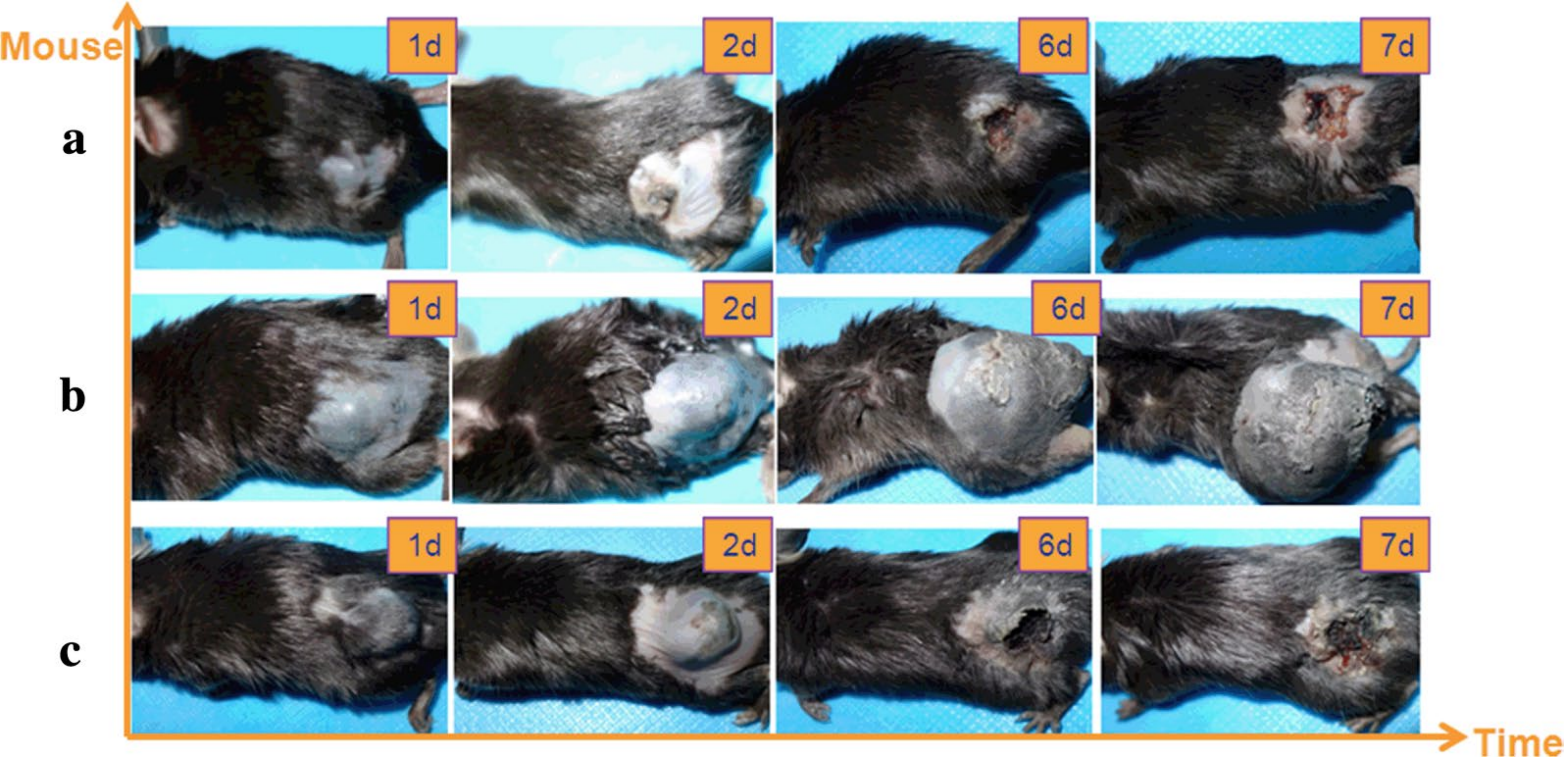
Pictures of electrical stimulation treatment of malignant melanoma tumor on mice model based on liquid metal spray-printing. **a** Electrical stimulation parameters of mice model (signal: sine wave, the voltage is 5 V, the electric field strength of about 2 V/cm, the frequency is 300 kHz). **b** Electrical stimulation parameters of mice model (signal: sine wave, voltage: 5 V, the electric field strength is about 2 V/cm, the frequency is 100 kHz). **c** Electrical stimulation parameters of mice model (signal: sine wave, voltage: 4 V, the electric field strength is about 1.6 V/cm, the frequency is 300 kHz)

Through in vivo treatment of tumors in C57BL/6 (B16F10 syngeneic tumor model), significant slowing of tumor growth and extensive destruction of tumor cells were obtained within 6 days. We found that 300 kHz of TTFields at low intensities of 5 V/cm effectively inhibited malignant melanoma growth compared with other electrical stimulation parameters. Photographs of examples of treated mice models with 300 kHz of TTFields at low intensities of 5 V/cm and other electrical stimulation parameters are given in (Fig. 2a–c) for comparison. Clearly, the sizes of the treated tumors were significantly smaller than that of control tumors at the end of treatment. In addition, total process was found to be both safe and effective in slowing tumor progression. These findings demonstrate the potential applicability of the described spray-printing TTFields as a novel therapeutic modality for tackling malignant tumors.

### Magnetic resonance imaging

**Fig. 3:**
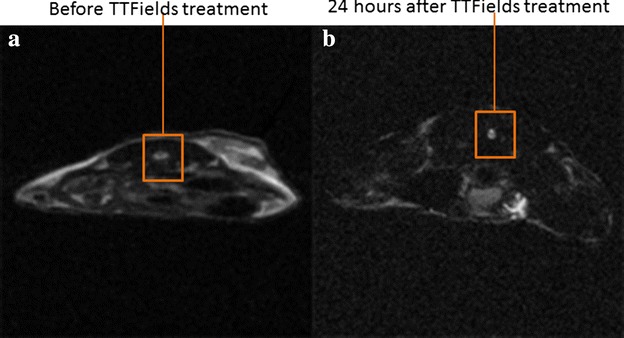
Magnetic resonance imaging (MRI). Exemplary partial radiological response to therapy in a representative mouse. **a** T1-weighted axial MRI images with pre-treatment (*left panel*). **b** 24 h after treatment (*right panel*)

Magnetic resonance imaging (MRI) data from a representative mouse that showed an almost complete response to the spray-printing TTFields treatment is shown in Fig. 3. It indicates that, a tumor was visible before TTFields treatment (Fig. 3a) while almost no tumor was detected 24 h after treatment (Fig. 3b). The tumor volume post-treatment was significantly smaller than that of the pre-treatment (23 versus 197 mm^3^) (Fig. 3a, b).

### Pathological results

Observed by naked eyes, after electrical stimulation therapy, in the experimental group mice (A) and (C), partial tumor becomes necrosis 2–3 days after applied TTFields treatment. And the tumors display central depression, with uneven surface. Then a week later, new tissue was seen around the necrotic tissue. In the (A) group, tumor group of mice after treatment gradually reduced within a week and finally disappeared. In the (C) group, tumor group was diminishing after 1 week of treatment, but more slowly. In the (B) group and the control group, the tumor of the mice continues to grow after treatment.

As the MRI image indicates (Fig. 3a, b), a tumor was visible before TTFields treatment (Fig. 3a) while almost no tumor was detected 24 h after treatment (Fig. 3b). The tumor volume post-treatment was significantly smaller than that of the pre-treatment (197 versus 23 mm^3^).

**Fig. 4:**
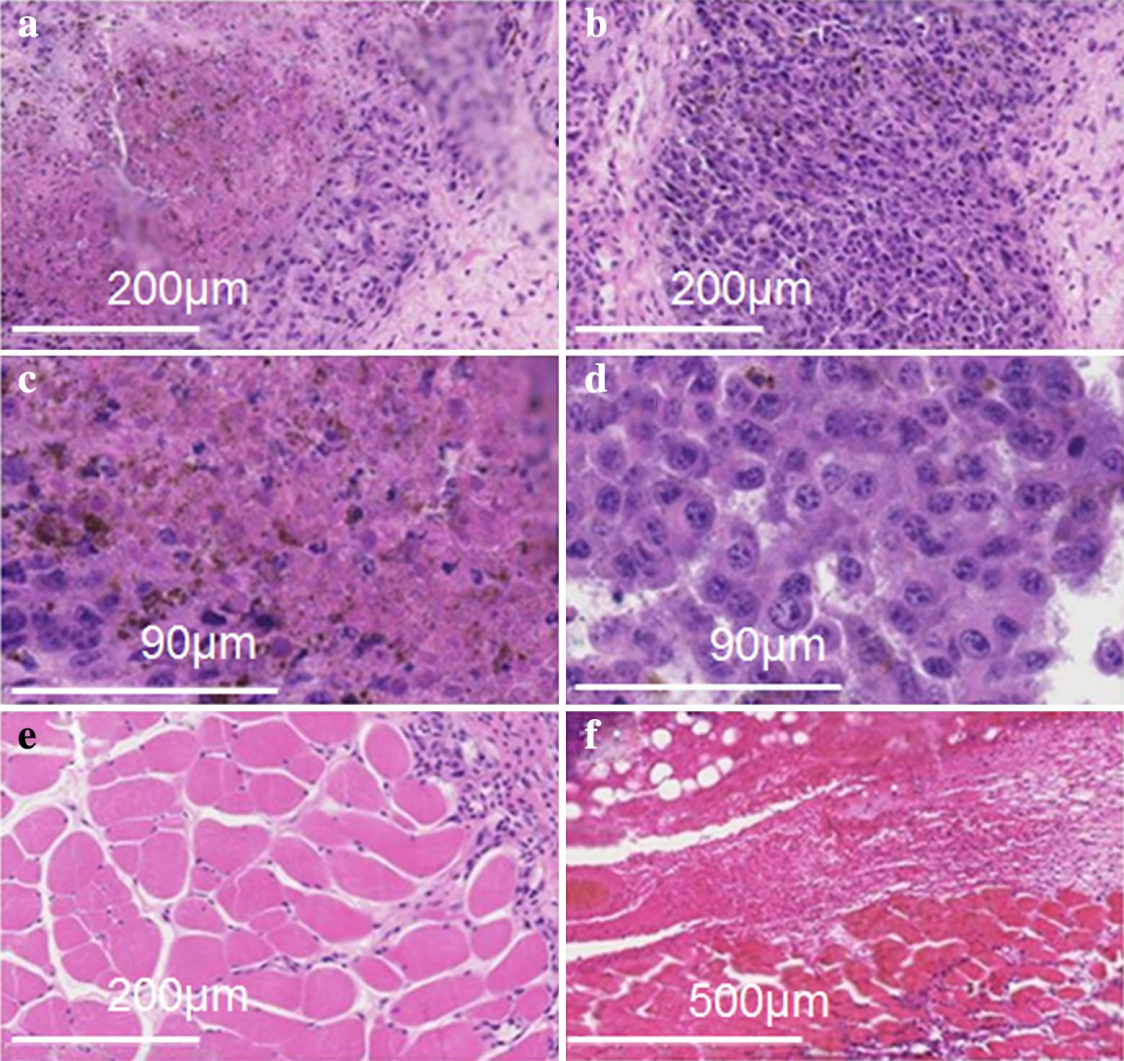
The pathological pictures as the mouse was subjected to electrical treatment three times after 72 h. **a** The picture for the case of most of the tumor cells was died. **b** The picture for the case of most of the tumor cells were apoptosis after electrical treatment based on liquid metal spray-printed electrodes. **c** The picture for the case of normal muscle tissues beside the tumor. **d** The enlarged picture where the tumor cells were apoptosis. **e** The enlarged picture of the tumor cells being died. **f** The section picture of tissue without tumor cells

In the (A) group, as shown in Fig. 4a, c, 24 h pathological examination demonstrated that a carbide changed the tumor tissue on the surface of the skin and the cancer nests on the integrity of the structure was completely destroyed.

**Fig. 5:**
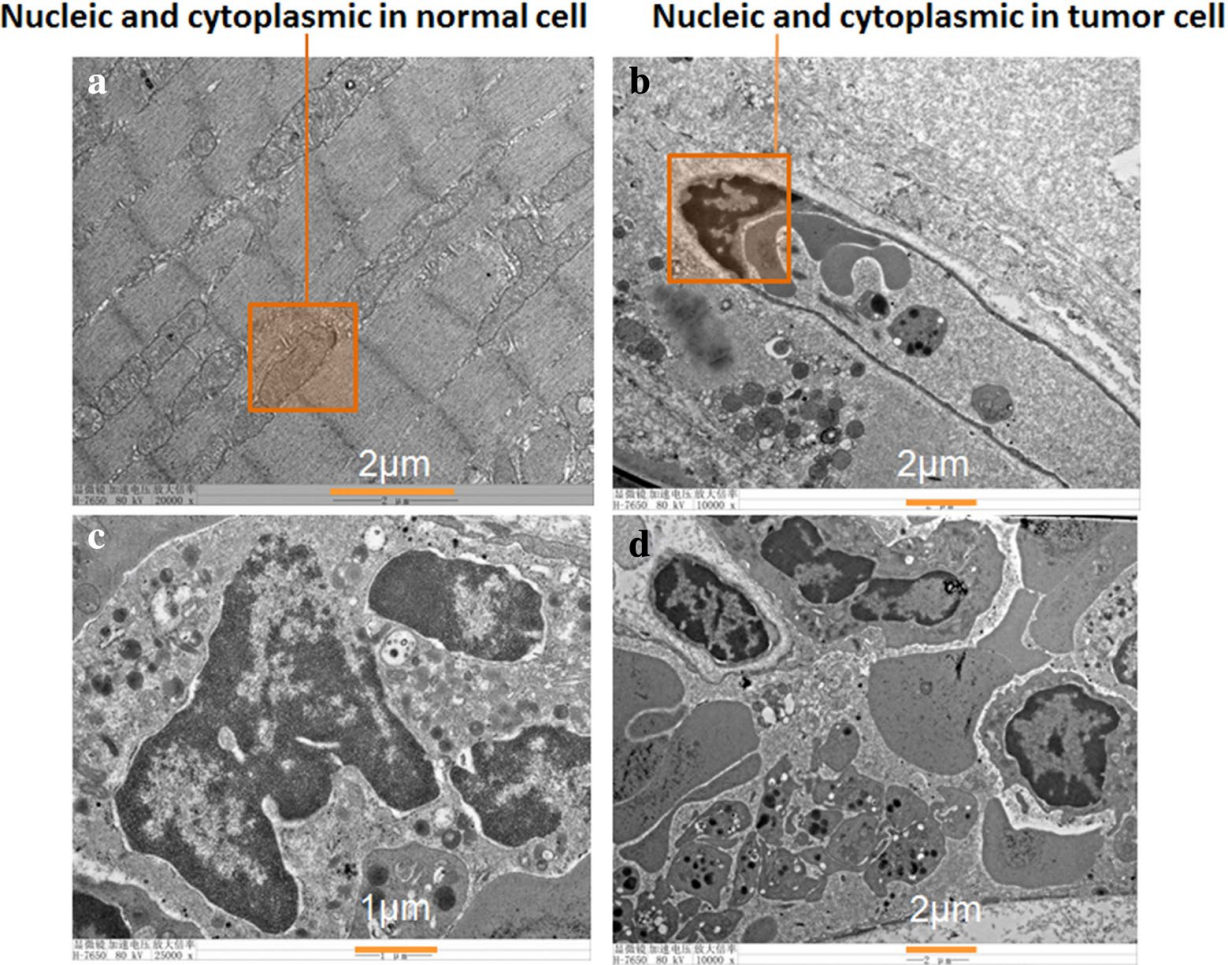
The SEM pictures as the mouse was electrically treated seven times after 168 h. **a** The picture of normal muscle tissues beside the tumor. **b** The picture where the tumor cell was died. **c** The picture where the nucleus of the tumor cell undergone fragmentation. **d** The picture where the fragmentation of the nucleus is outside of cell

After treatment, the tumors and their adjacent tissues were fixated, stained with H&E and analyzed histopathologically. Histopathological analysis of treated tumors showed extensive necrotic tissues and cellular debris. No damage to the surrounding tissues was detected (Fig. 4a–f). As the SEM figure indicates (Fig. 5a–d), there were no damage to the nucleus in normal cells, but the nucleic and cytoplasmic contents were losing in tumor cells. It can be explained that this treatment has produced good output regarding targeted therapy.

### Transmission electron microscopic test

To confirm the mouse mode of tumor cell death induced by spray-printing TTFields treatment, we performed further transmission electron microscopic tests. Necrotic cell death manifested by rupture of the plasma membrane and loss of nuclear and cytoplasmic contents was readily detected using transmission electron microscopy in post-treatment tumor. In contrast, tissues beside the tumor (muscle) resembled those untreated normal control, displaying intact plasma membrane and preserved nuclear architecture (Fig. 5a–d). Thus, spray-printing TTFields treatment induces necrosis in melanoma cells only.

### Thermal imaging images

**Fig. 6:**
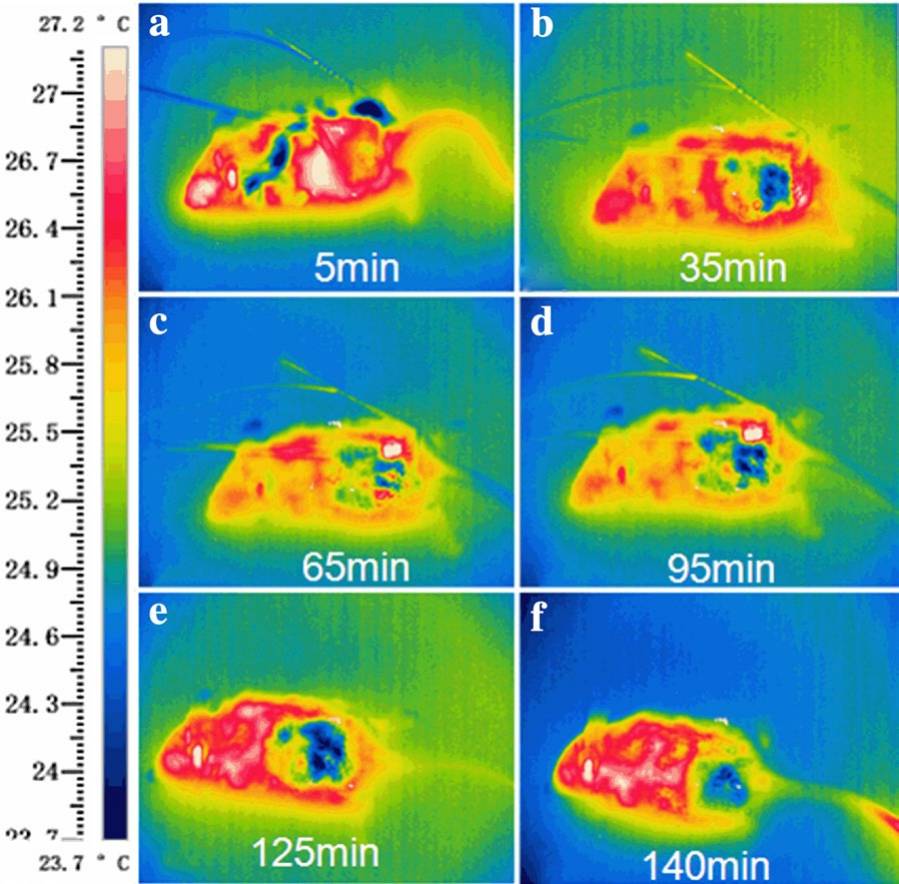
Thermal infrared images as the mouse was electrically treated from 5 to 140 min under anesthesia. **a** It is the 5th min before the spray-printing of liquid metal on the back of mouse with the 5 V voltage, 300 kHz sine wave electrical signal. **b**–**d** It is the 35th, 65th, 95th min after the spray-printing of liquid metal on the back of mouse with the 5 V voltage, 300 kHz sine wave electrical signal respectively. **e**, **f** It is the 125th, 140th min, after the end of electrical treatment and liquid metals were removed by medical alcohol

To exclude the temperature effect in our experiments, infrared camera (FLIR, US) was used to monitor the temperature change on the skin surface above the treated tumors in vivo, which showed no significant elevation in temperature during the period of treatment compared with pre and post-treatment (Fig. 6a–f). The results validate the existence of a non-thermal effect during spray-printing TTFields treatment.

### Discussion and conclusion

Traditionally, oncology therapy includes chemotherapy, surgery and radiation, or a combination thereof. More recently, molecularly targeted therapy and vaccines have held great promise for prolonging survival and, in some cases, preventing cancer. Physical modalities, other than ionizing radiation and heating, have been systematically studied as cancer treatment in just the past decade. Electric fields, while being utilized in various fields of medicine, had fallen into this category until very recently. In the past decade, antimitotic, frequency-tuned electric field therapy (low-intensity, intermediate-frequency alternating fields), also referred to as TTFields, have been studied and represent a promising regional oncology therapy.

Tumor treating fields is a noninvasive, regional antimitotic treatment modality that delivers low-intensity (1–3 V/cm), intermediate-frequency (100–300 kHz) and alternating electric fields to the tumor using transducer arrays placed on the skin around the region of the body containing the tumor. TTFields has been approved for the treatment of recurrent glioblastoma by the US. FDA and has a CE mark in Europe [16]. TTFields therapy affects metaphase, by disrupting mitotic spindle formation and anaphase, by dielectrophoretic dislocation of intracellular constituents, resulting in apoptosis. TTFields therapy relies on frequency tuned to specific cancer cell types. The antimitotic effect of TTFields therapy has been demonstrated in multiple cell lines when the appropriate frequency was utilized. Compared with active chemotherapy, TTFields therapy is associated with minimal toxicity, better quality of life and comparable efficacy in recurrent glioblastoma patients. Preclinical data demonstrate that the antimitotic effect of TTFields is intensity and frequency dependent and has additive or synergistic activity with chemotherapy [17–19]. In addition, TTFields therapy has been shown to inhibit systemic metastases in animal models [20]. Clinical data demonstrate comparable efficacy in recurrent glioblastoma with a more favorable safety profile and better quality of life than chemotherapy [with US Food and Drug Administration (FDA) approval in 2011].

But no matter whether they are solid electrode or the newly emerging skin-electronic devices, they do not directly contact with the skin indeed due to large contact resistance. This may generate strong coupling impedance with the skin. From an alternative way, the present work found a highly efficient method to directly pattern liquid metal conductive components as electrodes on skin through spray-printing strategy. This quick forward way of making flexible electronics on skin is enabled via stainless mask which is pre-designed via chemical etching with line width resolution of 100 µm and can be used to deposit desired electrical components [15].

In the present work, we have shown that spray-printing TTFields could evidently inhibit the growth of tumors in mice while causing no general side effects or local histopathological damage. This demonstrated the strategy’s inhibitory effect in all proliferating cell types as tested, whereas, nonproliferating cells and tissues were unaffected. Temperature measurements on the skin above treated tumors in vivo, showed no significant elevation in temperature. This indicates that this effect is nonthermal.

Our method of directly printing electronics pattern TTFields therapy owns the potential to completely change the treatment paradigm for solid tumor oncology, as a regional therapy that can encompass entire body regions, such as brain, lungs, or liver, which are the most common sites of solid tumor metastasis and the most common cause of mortality once involved. Meanwhile, the directly printed electronics pattern TTFields cancer treatment modality avoids the conventional cytotoxic systemic side effects and may enable chronic and continuous cancer therapy. Previous attempts to increase cell killing with chemotherapy through increasing dose density had limited success due to the cumulative toxicities of high-dose chemotherapy and radiation doses are limited due to potential damage to surrounding normal tissues.

Combination of our directly printing electronics pattern TTFields therapy with chemotherapy (surgery, radiation) may benefit from several ways: (1) Enhanced cell killing by chemotherapy due to a potential synergistic effect; (2) Continuous cytoreductive effect by TTFields therapy in between chemotherapy infusions to minimize tumor regrowth, DNA repair and emergence of drug resistance; (3) Continuous cytoreductive effect by TTFields therapy in between lines of chemotherapy; (4) Absence of overlapping toxicity; and (5) Inhibiting the metastasis. Together, these may add up to an exponential increase in cell killing, possibly sufficient to result in long-term tumor regression and control.

Compared with the conventional TTFields therapy, the current liquid metal electrode increased the contact area with the skin, enhanced therapeutic effects and reduced conventional TTFields adverse effects. The advantages of this liquid metal electrode TTFields therapy is expected to increase acceptability and is easy for patient use in the near future. Optimization of the field-generating device in terms of size, weight and longer battery life may enable long-term carrying and continuous cancer therapy throughout the day.
